# Promising Prebiotic Candidate Established by Evaluation of Lactitol, Lactulose, Raffinose, and Oligofructose for Maintenance of a Lactobacillus-Dominated Vaginal Microbiota

**DOI:** 10.1128/AEM.02200-17

**Published:** 2018-02-14

**Authors:** Stephanie L. Collins, Amy McMillan, Shannon Seney, Charlotte van der Veer, Remco Kort, Mark W. Sumarah, Gregor Reid

**Affiliations:** a Department of Microbiology and Immunology, The University of Western Ontario, London, Ontario, Canada; b Department of Surgery, The University of Western Ontario, London, Ontario, Canada; c Canadian Centre for Human Microbiome and Probiotics, Lawson Health Research Institute, London, Ontario, Canada; d Public Health Laboratory, Public Health Service of Amsterdam (GGD), Amsterdam, The Netherlands; e Molecular Cell Physiology, Faculty of Earth and Life Sciences, VU University, Amsterdam, The Netherlands; f Netherlands Organisation for Applied Scientific Research (TNO), Microbiology and Systems Biology, Zeist, The Netherlands; g Micropia, Natura Artis Magistra, Amsterdam, The Netherlands; h Agriculture and Agri-Food Canada, London, Ontario, Canada; Centers for Disease Control and Prevention

**Keywords:** bacterial vaginosis, lactitol, lactobacilli, lactulose, oligofructose, prebiotics, raffinose, vaginal microbiota

## Abstract

Perturbations to the vaginal microbiota can lead to dysbiosis, including bacterial vaginosis (BV), which affects a large portion of the female population. In a healthy state, the vaginal microbiota is characterized by low diversity and colonization by Lactobacillus spp., whereas in BV, these species are displaced by a highly diverse population of bacteria associated with adverse vaginal health outcomes. Since prebiotic ingestion has been a highly effective approach to invigorate lactobacilli for improved intestinal health, we hypothesized that these compounds could stimulate lactobacilli at the expense of BV organisms to maintain vaginal health. Monocultures of commensal Lactobacillus crispatus, Lactobacillus vaginalis, Lactobacillus gasseri, Lactobacillus johnsonii, Lactobacillus jensenii, and Lactobacillus iners, in addition to BV-associated organisms and Candida albicans, were tested for their ability to utilize a representative group of prebiotics consisting of lactitol, lactulose, raffinose, and oligofructose. The disaccharide lactulose was found to most broadly and specifically stimulate vaginal lactobacilli, including the strongly health-associated species L. crispatus, and importantly, not to stimulate BV organisms or C. albicans. Using freshly collected vaginal samples, we showed that exposure to lactulose promoted commensal Lactobacillus growth and dominance and resulted in healthy acidity partially through lactic acid production. This provides support for further testing of lactulose to prevent dysbiosis and potentially to reduce the need for antimicrobial agents in managing vaginal health.

**IMPORTANCE** Bacterial vaginosis (BV) and other dysbioses of the vaginal microbiota significantly affect the quality of life of millions of women. Antimicrobial therapy is often poorly effective, causes side effects, and does not prevent recurrences. We report one of very few studies that have evaluated how prebiotics—compounds that are selectively fermented by beneficial bacteria such as Lactobacillus spp.—can modulate the vaginal microbiota. We also report use of a novel *in vitro* polymicrobial model to study the impact of prebiotics on the vaginal microbiota. The identification of prebiotic lactulose as enhancing Lactobacillus growth but not that of BV organisms or Candida albicans has direct application for retention of homeostasis and prevention of vaginal dysbiosis and infection.

## INTRODUCTION

In most healthy reproductive-age women, Lactobacillus spp. are the main constituents of the vaginal microbiota. Their ability to acidify this niche through lactic acid production (1) and to otherwise modulate the environment (2) is profoundly important for female health and reproduction (3). However, in a condition known as bacterial vaginosis (BV), anaerobes such as Gardnerella vaginalis, Atopobium vaginae, Prevotella spp., and Mobiluncus spp. proliferate, sometimes causing aberrant symptoms and signs (4, 5). BV is commonly associated with an increase in odor-causing metabolites such as putrescine and cadaverine, as well as with the presence of gamma-hydroxybutyrate (GHB) (6). Furthermore, the displacement of Lactobacillus spp. produces elevated vaginal pH and predisposes women to pelvic inflammatory disease, HIV-1 transmission, and infection by Trichomonas spp., Chlamydia trachomatis, and Neisseria gonorrhoeae (7–9). Recurrent BV patients also have higher rates of vulvovaginal candidiasis, which is caused by overgrowth of the opportunistic Candida albicans (10). This condition is extremely common, with a reported prevalence of 30% in the United States (11), causing a significant burden to both women and the health care system (12).

The treatment for BV has for decades been administration of metronidazole or clindamycin targeting anaerobes. However, in addition to side effects and failure to restore microbiota homeostasis, large numbers of women fail to respond or relapse within months of treatment (13, 14). While some women maintain a stable vaginal microbiota, most show fluctuating patterns due to antimicrobial agents, menstruation, and sexual activity, as well as unknown factors (15). Too often, restoration of a Lactobacillus-dominant microbiota does not occur. This has led to interventions containing probiotic therapies, such as Lactobacillus reuteri RC-14 and Lactobacillus rhamnosus GR-1, which help the indigenous lactobacilli recover (16–18).

Another approach worth considering is the use of a prebiotic, which is defined as a substrate that is selectively used by beneficial host microorganisms to confer a health benefit (19). These are generally food-grade compounds, including inulin and other fructooligosaccharides (FOS), sugar alcohols, galactooligosaccharides (GOS), lactulose, and raffinose. They are known to stimulate native bifidobacteria and lactobacilli in the intestine, leading to a range of favorable gastrointestinal outcomes, including reduced inflammation (20). The concept of using selective nutrients to supplement vaginal microbes was first introduced by our group in 1995, when we showed that skim milk improved vaginal Lactobacillus numbers in women with recurrent urinary tract infections (21). Several prebiotics, including GOS (22, 23), FOS (23, 24), and glucomannan hydrolysates (25, 26), have since been evaluated for their efficacy in stimulating vaginal lactobacilli in both *in vitro* and small-scale human trials. The assessment of these and other formulations, however, has been based on testing against single strains or on characterizing dysbiosis using combinations of culture- and microscopy-based techniques, which lack the sensitivity needed to detect the numerous low-abundance anaerobes present in BV. For example, organisms designated BVAB1 to BVAB3 have been deemed critical for BV onset and pathogenesis, yet have been unculturable to date (27–29). Instead, molecular techniques such as 16S rRNA gene sequencing and metabolomics (used in this study) should be adopted to accurately observe modifications to the vaginal microbiota.

Therefore, the objective of the present study was to develop a method for testing the effects of prebiotic compounds against a range of biologically relevant vaginal lactobacilli and bacteria associated with dysbiosis, and to apply 16S rRNA gene sequencing and mass spectrometry-based metabolomics to assess their impact.

## RESULTS

### Fermentation profiles of prebiotics by vaginal microbes.

**FIG 1:**
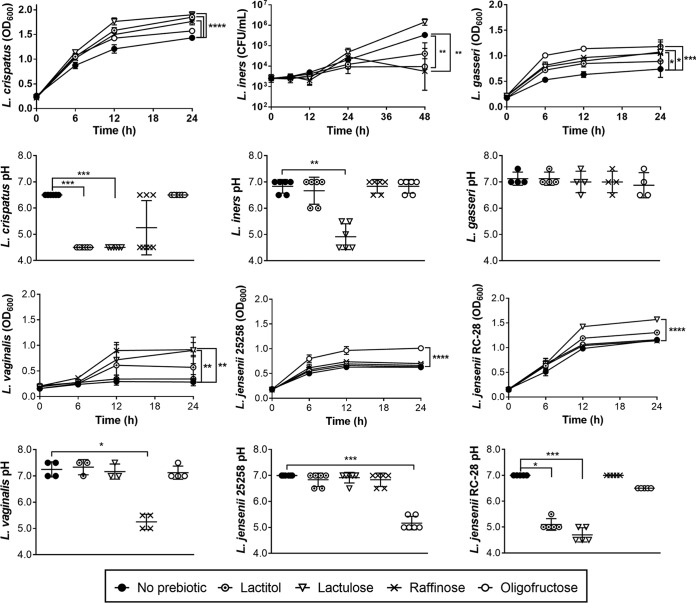
Growth and pH of vaginal lactobacilli cultured in prebiotics. Individual cultures of vaginal lactobacilli inoculated in 0.5% prebiotic media prior to pH testing. For growth curves, points represent mean (optical density at 600 nm [OD_600_]) or geometric mean (CFU/ml) of 3 to 7 replicates ± standard error of the mean (SEM), with differences determined using 2-way ANOVA with Dunnett's multiple-comparison test. For pH measurements, bar heights are mean pH ± SD, with differences from control determined using Kruskal-Wallis' with Dunn's multiple comparison tests.

**FIG 2:**
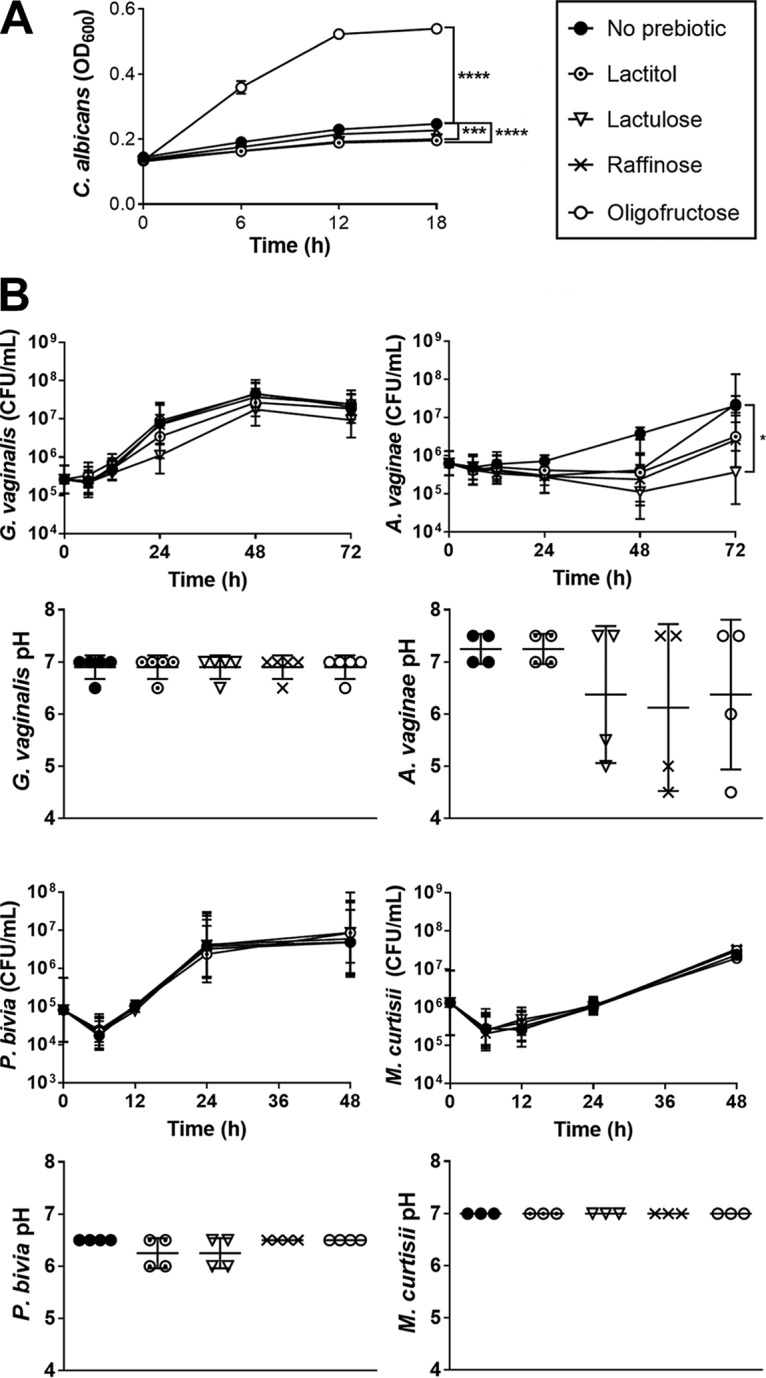
Growth and pH of C. albicans and BV organisms cultured in prebiotics. (A) C. albicans supplemented with 5% prebiotic. (B) BV-associated bacteria inoculated in 0.5% prebiotic media prior to pH testing. For growth curves, points represent mean (OD_600_) or geometric mean (CFU/ml) of 3 to 7 replicates ± SEM, with differences determined using 2-way ANOVA with Dunnett's multiple-comparison test. For pH measurements, bar heights are mean pH ± standard deviation (SD), with differences from control determined using the Kruskal-Wallis test with Dunn's multiple comparison test.

Following bacterial culture in 0.5% prebiotic-supplemented dextrose-free media, pH was measured postincubation to indicate whether prebiotics were fermented and if this process could restore acidity, an essential component of vaginal health. Lactulose increased the maximal growth of Lactobacillus crispatus (*P* < 0.0001), Lactobacillus gasseri (*P* < 0.05), Lactobacillus vaginalis (*P* < 0.01), and Lactobacillus jensenii RC-28 (*P* < 0.0001) compared to that in media without prebiotic (Fig. 1). Lactulose treatment also correspondingly lowered the pH compared to that in media without prebiotic for L. crispatus (*P* < 0.001) and L. jensenii RC-28 (*P* < 0.001) (Fig. 1). Although lactulose did not significantly elevate the viable counts of Lactobacillus iners (*P* = 0.50), the pH was lowered compared to that in media without prebiotic (*P* < 0.01) (Fig. 1). This suggests that L. iners produces fermentation products upon lactulose inoculation, despite not growing. Lactulose limited the growth of C. albicans (*P* < 0.001) and A. vaginae (*P* < 0.05) compared to media without prebiotic, while not affecting the growth of G. vaginalis, Prevotella bivia, or Mobiluncus curtisii (Fig. 2). Overall, lactulose was broadly and effectively used by vaginal Lactobacillus spp., while not stimulating potential pathogens.

Since an L. crispatus-dominated vaginal microbiota is most strongly correlated with positive health outcomes (15, 30), and strains of the same species can vary greatly in their ability to degrade sugars, the metabolism of lactulose by 15 clinical L. crispatus isolates was also evaluated. Despite being extracted from healthy and BV-affected women, all L. crispatus isolates were stimulated by lactulose treatment (see Fig. S1 in the supplemental material).

Oligofructose, raffinose. and lactitol were less preferred than lactulose by vaginal lactobacilli in culture. Oligofructose improved the growth of L. gasseri (*P* < 0.001) and L. jensenii 25258 (*P* < 0.0001) compared to that in the control without prebiotic, although acidic pH was only reached by L. jensenii 25258 (*P* < 0.001) (Fig. 1). However, oligofructose antagonized L. iners (*P* < 0.01) (Fig. 1) and attenuated the growth of C. albicans compared to that in media lacking prebiotic (*P* < 0.0001) (Fig. 2A). Raffinose elevated the maximal growths of L. crispatus (*P* < 0.0001), L. gasseri (*P* < 0.05), and L. vaginalis (*P* < 0.01), causing medium acidification only in L. vaginalis culture (*P* < 0.05) (Fig. 1). Conversely, raffinose reduced L. iners viability compared to that in media without prebiotic (*P* < 0.01) (Fig. 1). Although lactitol only stimulated L. crispatus growth (*P* < 0.0001), acidic products were produced by both lactitol-inoculated L. crispatus (*P* < 0.001) and L. jensenii RC-28 (*P* < 0.05) (Fig. 1). Furthermore, lactitol reduced C. albicans density relative to that in media without prebiotic (*P* < 0.0001) (Fig. 2A). No notable growth of G. vaginalis, A. vaginae, M. curtisii, or P. bivia was observed in medium containing oligofructose, raffinose, or lactitol (Fig. 2B).

### Bacterial composition of vaginal samples incubated with prebiotics.

**FIG 3:**
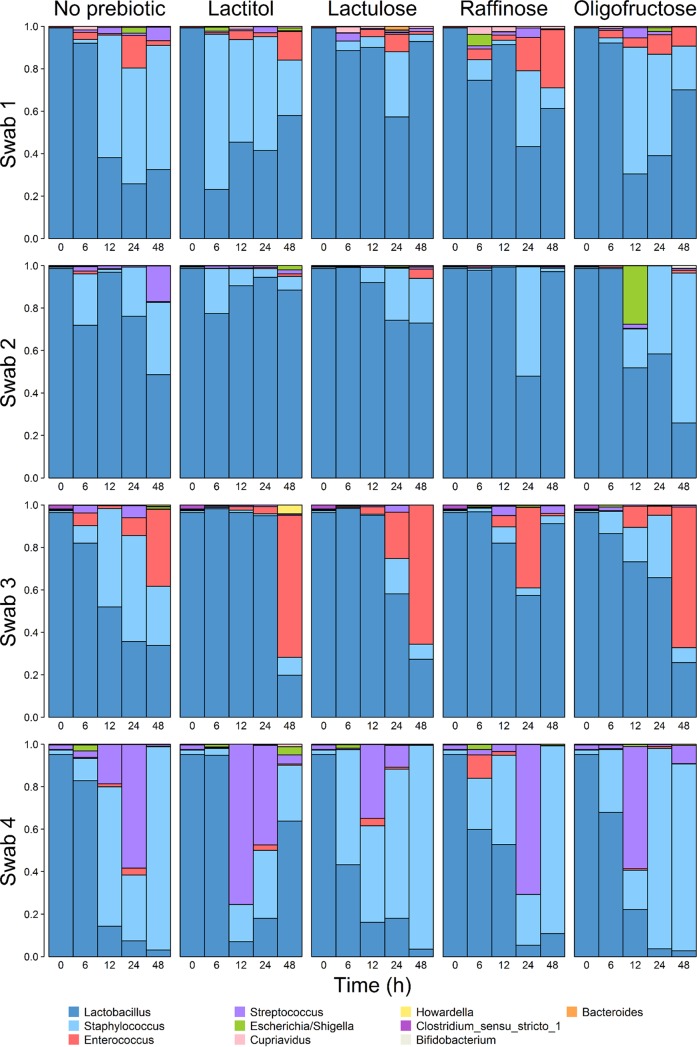
Swab microbiota dynamics throughout inoculation in prebiotics. Vaginal swab microbiota from four separate donors were cultured in dextrose-free vaginally defined medium with 0.5% (wt/vol) prebiotic. Bar heights represent the fraction of each genus at each sampling point, as determined by 16S rRNA gene sequencing of the V4 region.

Having established that lactulose most effectively stimulated Lactobacillus spp. in monoculture, we developed a method to culture the community of vaginal microbiota from swabs collected from healthy women. These swabs were then inoculated in media containing 0.5% prebiotic or in control media without prebiotic. Initially, all four healthy swabs were dominated by Lactobacillus spp., with those from subjects 1, 3, and 4 mostly composed of L. crispatus, and that of subject 2 was mostly composed of L. iners (Fig. 3). The microbial community of each subject reacted distinctly to prebiotic treatment. Notably, each prebiotic appeared to improve the resilience of lactobacilli. In sample 1, by 48 h lactobacilli increased in abundance in each prebiotic relative to that in the control media (Fig. 3). Lactulose in particular maintained Lactobacillus dominance, as noted by its restoration to above 90% abundance by 48 h (Fig. 3). In sample 2, lactulose, as well as lactitol and raffinose, improved Lactobacillus maintenance, while oligofructose supplementation caused a relative abundance shift toward Staphylococcus spp. dominance compared to the abundance in the control without prebiotic (Fig. 3). Sample 3 microbiota responded similarly to treatment with lactulose, lactitol, and oligofructose, with early time points showing elevated Lactobacillus abundance compared to that in the control before 48 h postinoculation (Fig. 3). Raffinose supplementation, on the other hand, improved Lactobacillus abundance throughout the 48-h period (Fig. 3). Only lactitol induced prolonged maintenance of Lactobacillus spp. in sample 4, compared to media without prebiotic and media supplemented with lactulose, raffinose, or oligofructose (Fig. 3).

**FIG 4:**
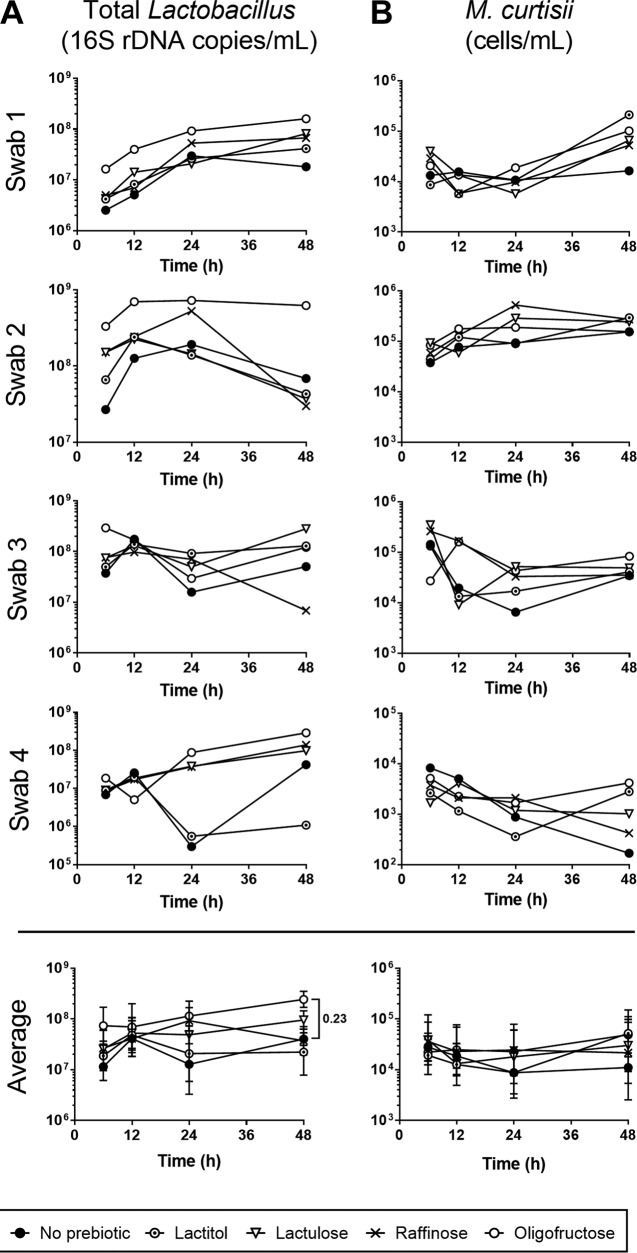
Growth of lactobacilli and M. curtisii in prebiotic-supplemented vaginal swab microbiota. Concentration of (A) Lactobacillus spp., and (B) M. curtisii 16S rRNA genes from vaginal swab microbiota grown in 0.5% prebiotic. Points represent individual measures of growth for each individual swab, and are the geometric mean ± SEM for the average of all swabs. Geometric means were analyzed for significant differences using two-way ANOVA with Dunnett's multiple-comparison test.

To elucidate whether these proportional changes were due to increased numbers of beneficial lactobacilli, rather than to a loss of aerobic organisms, we next used quantitative PCR (qPCR) for total Lactobacillus numbers. Lactulose treatment increased Lactobacillus abundance by 48 h in L. crispatus-dominant samples 1, 3, and 4 and at early time points for sample 2 (Fig. 4A). Oligofructose consistently stimulated lactobacilli, increasing their abundance in all four swabs after 48 h, although the mean change was not significant (*P* = 0.2388) (Fig. 4A). Raffinose slightly increased total lactobacilli after 48 h in samples 1 and 3 while decreasing their numbers in samples 2 and 4, compared to those in media without prebiotic (Fig. 4A). Lactitol increased Lactobacillus levels in samples 1 and 4 while decreasing them in samples 2 and 3 (Fig. 4A).

Since BV-related organisms were in low relative abundance and therefore were not observable in the context of the whole microbiota, their abundances as a centered log ratio were monitored individually. Notably, none of the detectable BV-associated organisms present in the swab communities were stimulated by prebiotic treatment (see Fig. S2 in the supplemental material). Two commonly characterized BV organisms, A. vaginae and M. curtisii, were not proportionally abundant enough to be identified by sequencing, so absolute quantification qPCR was adopted to specifically target these bacteria. In further support of its absence, A. vaginae was not detected with this technique (data not shown). M. curtisii levels were elevated to the greatest extent by lactitol and oligofructose in samples 1 and 4, although neither reached significance overall (Fig. 4B).

### Metabolite changes in prebiotic-cultured vaginal swab communities.

**FIG 5:**
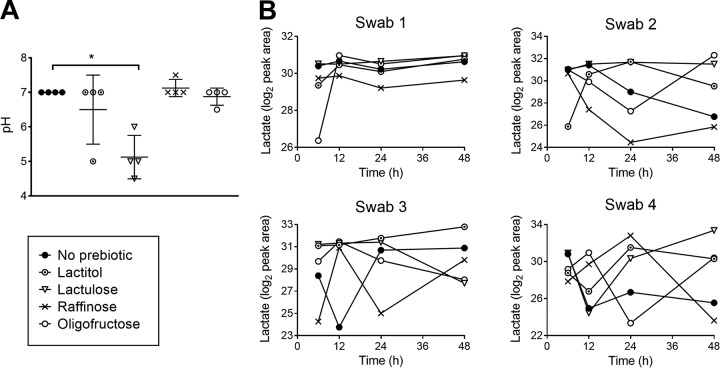
Metabolic analysis of vaginal swab consortia in prebiotics. (A) Mean pH ± SD of swab communities following 48 h of growth in prebiotics. Statistical significance determined according to the Kruskal-Wallis test with Dunn's multiple comparison test (*, *P* < 0.05). (B) Peak area of LC-MS (liquid chromatography-mass spectrometry)-detected lactate, log 2 corrected, in swab bacterial supernatant. Dots represent individual measures of growth over time.

Several other important indicators of vaginal health include acidity and metabolites from actively fermenting commensal lactobacilli (31, 32). To determine whether acidity could be restored by prebiotic treatment in the healthy vaginal microbiota model, swab samples were pH tested following 48 h of incubation with the prebiotics. Lactulose was found to significantly lower the pH (*P* < 0.05), a phenomenon not observed with any other prebiotic (Fig. 5A). Since *in vivo* vaginal acidification in healthy women is predominantly due to lactate production by lactobacilli (33), liquid chromatography-mass spectrometry (LC-MS) quantification of this metabolite was performed. After 48 h, lactulose and oligofructose increased lactate production in the cultures of samples 1, 2, and 4 compared to that in media without prebiotic but only increased lactate at early time points in sample 3 (Fig. 5B). Lactitol also increased lactate abundance in samples 2, 3, and 4, while raffinose lowered medium lactate compared to that in control with no prebiotic in samples 1, 2, and 3 (Fig. 5B).

To confirm that prebiotic utilization by bacteria in the human samples was the cause of medium acidification and lactate production, gas chromatography-mass spectrometry (GC-MS) of spent media was used to detect levels of prebiotics following incubation. Both lactulose (*P* < 0.05) and lactitol (*P* < 0.01) were significantly lowered in the media following 48 h of swab bacterial growth. Conversely, raffinose levels remained static (see Fig. S3 in the supplemental material), indicating that changes caused by raffinose treatment were not due to bacterial metabolism of this prebiotic. Oligofructose's highly polymerized and variable structure prevented accurate detection of this prebiotic, and therefore it was eliminated from analysis.

Since GHB and 2-hydroxyisovalerate are specific biomarkers of BV, and cadaverine, putrescine, and tyramine are responsible for vaginal odor (6, 34), we measured these metabolites in the vaginal samples inoculated with prebiotics, and we did not detect them under any growth condition (data not shown).

## DISCUSSION

Herein, we have characterized a candidate prebiotic, lactulose, for its ability to promote microbial and metabolic homeostasis in a healthy vaginal microbiota model, independent of antibiotic use. We showed that supplementation of bacterial monocultures with lactulose stimulated L. crispatus, L. gasseri, L. vaginalis, and L. jensenii RC-28, while antagonizing C. albicans and A. vaginae, indicating its ability to selectively propagate a range of commensals but not vaginal pathogens. Furthermore, in the case of L. crispatus, bacterial stimulation with lactulose was not strain-specific, indicating that this prebiotic could have applications in many women. Although lactulose did not improve the viability of L. iners, the drastically lowered medium pH suggests that lactulose was fermented by L. iners to produce acidic metabolites (Fig. 1). These effects were confirmed in vaginal samples from healthy women.

The lowering of pH reflects the increased production from lactulose of the fermentation byproduct lactic acid. Importantly, lactic acid and low pH are indicative of vaginal health, and they inhibit the development of BV and other dysbioses (32, 35, 36). Lactulose has also been reported in previous studies to rapidly increase medium acidity in Lactobacillus cultures (37, 38), suggesting that it might provide an immediate advantage to acid-tolerant, commensal lactobacilli over pathogens. Combined with the fact that lactulose was spent from the media during swab incubation, indicating its usage by the present microbes, this prebiotic has the greatest support for use as a vaginal prebiotic.

The model developed herein for studying the effects of prebiotics on the vaginal microbiota uses the organisms already active within the vaginal ecosystem of each subject. Although less reproducible than a defined steady-state culture, utilization of samples taken from numerous donors allows improved insight into the breadth of responses that could be observed in vaginal microbiota interventions. As we observed here, despite genus-level similarities in microbiota composition, the selective nature of prebiotics exerts variable effects on bacterial proportions because of differences in bacterial expression of carbohydrate metabolism genes, even at the strain level (39, 40). As any candidate intervention must eventually be applicable to women, a method that more closely predicts diversity in responses has merit. Ultimately, lactulose must be taken into a randomized clinical trial to prove its functionality in humans.

One drawback is that the artificial vaginal medium is not selective against aerobes such as Staphylococcus spp. and Enterococcus spp. Since these organisms are not often colonizers of the vaginal niche and are likely only present as a sampling artifact, this does not reflect *in vivo* efficacy of the tested compounds.

Of the other prebiotic compounds assessed, lactitol was also fermented by vaginal lactobacilli in monoculture, and it increased the proportional abundance of Lactobacillus spp. in the vaginal sample consortium. However, despite an increase in medium lactate, this compound did not restore an acidic pH. This suggests that the quantity of lactate produced by the lactitol-treated consortium was not sufficient to lower medium pH. In practice, lactitol could be administered with pH-lowering reagents in a two-pronged approach, a concept that has been taken into product development but not yet evaluated. Lactitol administered with lactobacilli following clindamycin treatment has been shown to improve recovery from BV (41), but the role of lactitol alone was not delineated, leaving questions about its contribution to this therapy.

In culture, raffinose was fermented by several Lactobacillus strains, but it appeared to antagonize L. iners, a common vaginal commensal found in women with and without BV (1, 42). Although raffinose positively altered the composition of the vaginal swab consortium, it did not directly stimulate lactobacilli. This suggests that raffinose inhibits the growth of bacteria such as Staphylococcus spp. or Enterococcus spp., thereby allowing commensal lactobacilli to propagate. This is further supported by the lack of fermentation products produced and the high levels of raffinose remaining in the spent media of the vaginal sample. However, other researchers have observed that raffinose can be used by Trichomonas vaginalis (43), the vaginal parasite responsible for trichomoniasis, which further underscores the unsuitability of this compound as an effective vaginal health agent.

Oligofructose was utilized by L. gasseri and L. jensenii 25258 in our study, and by Lactobacillus acidophilus in a previous investigation (24). However, despite its Lactobacillus-stimulating properties in our vaginal microbiota model, oligofructose did not increase the proportion of lactobacilli, and it antagonized L. iners AB-1 in culture. Oligofructose also stimulated growth of C. albicans, the causative agent of vulvovaginal candidiasis, and it did not acidify the swab sample despite doing this in monoculture. Since polymerized oligosaccharides, such as oligofructose, tend to be fermented more slowly and to a lesser degree than short oligosaccharides (44), it may have taken longer than the allotted 48 h to produce sufficient metabolic products to lower the pH. It is important to note that the retention time of prebiotics in the vagina is likely much shorter than colonic transit time, and therefore quick-releasing effects would be optimal for this niche. Others have observed that FOS with lower degrees of polymerization than oligofructose are used by vaginal Lactobacillus isolates and not by C. albicans or G. vaginalis (23); thus, this does not rule out all oligosaccharides for vaginal application. However, only a single strain of each Lactobacillus was tested in monoculture in these studies, so the effect of oligosaccharides on a complex culture system remains to be investigated. For the reasons stated above, our results suggest against the use of oligofructose for vaginal health.

In summary, we have demonstrated that lactulose has the potential to be a prebiotic, as it selectively stimulates vaginal lactobacilli, but not pathogenic or dysbiotic organisms, and promotes a healthy acidic environment. Confirmation in this novel human model provides a basis for further exploration of lactulose in a clinical trial for maintenance of vaginal eubiosis. This application is broadly applicable, due to the frequent microbial fluctuations women experience throughout the menstrual cycle (45) and from sexual activity (46, 47). Another forthcoming potential use for lactulose may involve conditioning an individual's own aberrant microbiota *ex vivo* for beneficial reintroduction into the vagina, a concept that has been called TripleA therapy (48). Ultimately, this inexpensive compound could reduce the necessity for antibiotics and avoid their side effects and inability to prevent dysbiotic recurrences.

## MATERIALS AND METHODS

### Strains, growth conditions, and prebiotics used.

Vaginal Lactobacillus strains (L. crispatus ATCC 33820^T^, L. vaginalis NCFB 2810, L. gasseri ATCC 33323^T^, L. jensenii RC28, and L. jensenii ATCC 25258) and Lactobacillus johnsonii ATCC 20553 were cultured anaerobically on MRS agar (Becton, Dickinson & Company, Sparks, MD) in jars at 37°C. L. iners AB-1 and the BV-associated bacteria Gardnerella vaginalis ATCC 14018, Atopobium vaginae ATCC BAA-55, Prevotella bivia ATCC 29303, and Mobiluncus curtisii ATCC 35241 were cultured anaerobically on Columbia agar with 5% sheep's blood (CBA) (Becton, Dickinson & Company) at 37°C in a controlled chamber (80% N_2_, 10% CO_2_, and 10% H_2_). Candida albicans TIMM 1768 was aerobically cultured on lysogeny broth (LB) agar at 37°C. Prebiotics selected were lactitol monohydrate (Alfa Aesar, Ward Hill, MA), lactulose (Alfa Aesar), d-(+)-raffinose (Sigma, St. Louis, MO), and Orafti P95 oligofructose (Beneo, Oreye, Belgium).

Measurements of optical density at 600 nm (OD_600_) were taken for Lactobacillus growth curves, excluding that of L. iners, in dextrose-free MRS with 0.5% (wt/vol) prebiotic using the Multiskan Ascent plate reader (Thermo-Fisher, Waltham, MA). Due to their inability to be cultured in a microaerophilic environment, growth of L. iners, G. vaginalis, A. vaginae, P. bivia, and M. curtisii was monitored using drop plating in an anaerobic chamber (80% N_2_, 10% CO_2_, and 10% H_2_) in dextrose-free New York City (NYC) III broth with 0.5% prebiotic. Growth was determined by comparison to control media without dextrose. Once the growth medium was spent, cultures were subjected to pH fermentation testing (VWR International, Radnor, PA). C. albicans viability in dextrose-free trypticase soy broth (TSB) with 5% prebiotic was measured by optical density during constant aerobic agitation at 37°C.

### Confirmatory analysis of clinical L. crispatus strains.

The clinical L. crispatus strains were isolated from vaginal swabs used to remove abundant mucus during routine cervical examinations at the sexually transmitted infections (STI) clinic in Amsterdam, the Netherlands. These swabs, which are normally discarded, were collected in June and July 2012 and immediately placed in transport media with additional 15% glycerol and stored at −80°C. As visitors to the STI clinic were informed that their samples may be used for scientific research after anonymization, and no extra clinical procedure was performed, no official approval under the Dutch Medical Research Involving Human Subjects Act (WMO) was required (reference number W12_86 12.17.0104). For this study, vaginal swabs from women with or without BV (according to the Nugent score) were plated on TSB agar supplemented with 5% serum and 0.25% lactic acid with pH set to 5.5 and grown under microaerobic conditions (6% O_2_) for 2 to 3 days (49). L. crispatus strains were identified based on colony morphology and their 16S rRNA gene sequence. To measure growth in lactulose, L. crispatus strains were inoculated as above in dextrose-free MRS supplemented with 0.5% (wt/vol) lactulose, and OD_600_ measurements were taken every 30 min for 24 h.

### Human subjects and vaginal swab collection.

Four healthy premenopausal women (aged 25 to 30 years) volunteered for the study, which was approved by the Health Sciences Research Ethics Board at Western University. After signing informed consent forms, women self-swabbed the lateral vaginal walls five times using separate sterile Dacron swabs. A healthy vaginal pH of <4.5 was confirmed by participants using pHem-Alert applicator keys (Gynex Corporation, Redmond, WA). Confirmation of healthy status was performed by smearing a swab on a microscopic slide and Gram staining (Becton, Dickinson & Company), and was scored according to the Nugent system (50). Subjects were included only if a normal Nugent score of 0 to 3 was observed, indicating the presence of large Gram-positive rods. Independent swabs were used for initial microbiota analysis and assessment of prebiotic effects over time.

### Incubation of vaginal samples in prebiotics.

Each vaginal swab was placed in 1 ml of phosphate-buffered saline (PBS) and vortexed for 5 min to release the bacteria and cellular contents. The liquid was then equally distributed into dextrose-free vaginally defined media + peptone (VDMP) (51) with or without 0.5% prebiotic. Individual time points and prebiotic growth conditions had separately designated tubes. Growth of swab bacteria was performed in anaerobic jars at 37°C until designated time points, when tubes were centrifuged for 10 min at 21,000 × *g* to separate cells from media. The pellet was subjected to microbiome analysis (see below), and supernatant was assessed for metabolites by mass spectrometry, and for acidification using pH 2.0 to 9.0 test strips (VWR).

### DNA extraction.

Microbial DNA was extracted from swab microbiota using the PowerSoil-htp 96-Well Soil DNA isolation kit (Mo Bio Laboratories, Carlsbad, CA) according to the manufacturer's instructions.

### Microbiota analysis by 16S rRNA gene sequencing.

The V4 variable regions of the 16S rRNA genes were amplified in a PCR mixture containing 50 μl light mineral oil, 1 μl sample DNA, and 10 μl each of barcoded primers at 3.2 pmol/μl, which together were set to 85°C before adding 20 μl of GoTaq master mix (Promega, Madison, WI). Reactions were primed at 95°C for 3 min, followed by 25 cycles of 95°C for 1 min, 52°C for 1 min, and 72°C for 1 min. Samples, including amplified no-template controls, were subsequently prepared and sequenced at the London Regional Genomics Centre (www.lrgc.ca; London, Ontario, Canada). First, DNA was quantified using a Qubit 2.0 fluorometer (Thermo-Fisher), pooled at an equal volume of each sample, and purified using the QIAquick PCR purification kit (Qiagen, Hilden, Germany). Purified amplicons were then paired-end sequenced with 250 cycles on an Illumina MiSeq platform (San Diego, CA) in 5% Phi X. A total of 1.91 × 10^7^ reads were obtained, with a median of 115,072 reads/sample.

The protocol for initial processing of reads was adapted from the dada2 workflow by Greg Gloor (github.com/ggloor/miseq_bin), using the dada2 and ShortRead packages in R version 3.2.2 (www.r-project.org). Read quality was determined by plotting quality profiles for both the forward and reverse reads. Trimming of reads was performed to remove primer barcodes and to remove low-quality ends of forward and reverse reads, which occurred at lengths of 183 and 174, respectively. Filtering was also performed to remove any reads with unidentified nucleotides and more than 2 expected errors, leaving 1.44 × 10^7^ reads with a median of 88,758 reads/sample. Dereplication was performed to summarize individual sequence units (ISUs) by abundance in each sample. Reads derived from PCR or sequencing errors were detected and removed using joint sample inference and error rate estimation on dereplicated ISUs. Pairs of forward and reverse reads were then overlapped and merged into complete sequences. By summarizing overlapping sequences by length, outliers were removed. Five 200-bp sequences, one 201-bp sequence, and two 209-bp sequences were identified and removed, leaving only sequences of 238 to 240 bp in length. Following chimeric sequence identification and removal, a final output of 139 unique sequences across all samples remained. These sequences and abundances can be found at https://figshare.com/s/f3c7c1ea91073f86d9ed (figshare.com). Taxonomy was generated to the genus level by comparison of best hits to the Silva rRNA database v123, and to the species level, where possible, according to the Silva species assignment database v123 (www.arb-silva.de). Taxonomy was designated when sequences matched the species with 100% identity, and there were no other matches above 97% identity. Operational taxonomic units (OTUs) were then created by grouping at the genus level. Only OTUs with an abundance of at least 1% across all samples were included for further analysis, leaving only 10 unique genera. To calculate centered log ratios (CLRs) for compositional analysis (52), zero-value OTU counts were replaced with an estimate value and then subjected to CLR calculation. Resulting CLRs were graphed in stacked bar plots using R software (www.r-project.org).

### Absolute quantification qPCR.

qPCR was performed on swab genomic DNA (gDNA) for 16S or 16S-23S rRNA genes of A. vaginae, G. vaginalis, L. crispatus, L. gasseri, L. iners, L. jensenii, L. vaginalis, and total Lactobacillus, and the mucin-desulfating sulfatase *mdsC* gene from P. bivia, as previously described (53, 54). Concentrations of forward and reverse primers in each reaction mixture were as follows: 200 nM (LgassF/LgassR, InersFw/InersRev, LV16s_23s_F/LV16s_23s_R3, M. curtisiiF/R, and PBsulF/PBsulR), 150 nM (LBF/LBR), 100 nM (LcrisF/LcrisR), 300 nM (LjensF/LjensR), 700 nM (AV-F/AV-R), 1,250 nM (F-GV1), and 625 nM (R-GV3). Reactions were performed in triplicate with 5 μl of 100-fold diluted gDNA, 5 μl of primers at their optimal concentration in double-distilled water (ddH_2_O), and 10 μl Power SYBR green PCR master mix (Life Technologies, Warrington, UK). Amplification was mediated by the 7900HT Fast real-time PCR system (Thermo), with cycling parameters indicated in Table 1. To detect primer dimers and other nontarget double-stranded DNA, cycling was followed by melt curve analysis. No significant contaminant annealing was detected (data not shown).

**TABLE 1:** Primer and thermal cycling parameters for RT-qPCR^*a*^

Target	Primer name	Primer sequence (5′ to 3′)	Thermal cycling parameters		
			Priming cycle	No. of cycles	Subsequent cycles
All Lactobacillus sp.	LBF	ATGGAAGAACACCAGTGGCG	15 min, 95°C	37	15 s 95°C, 45 s 50°C, 45 s 72°C
	LBR	CAGCACTGAGAGGCGGAAAC			
L. iners	InersFw	GTCTGCCTTGAAGATCGG	15 min, 95°C	35	15 s 95°C, 55 s 60°C, 60 s 65°C
	InersRev	ACAGTTGATAGGCATCATC			
L. crispatus	LcrisF	AGCGAGCGGAACTAACAGATTTAC	15 min, 95°C	40	15 s 95°C, 60 s 60°C, 20 s 72°C
	LcrisR	AGCTGATCATGCGATCTGCTT			
L. gasseri	LgassF	AGCGAGCTTGCCTAGATGAATTTG	15 min, 95°C	40	15 s 95°C, 60 s 57°C, 60 s 65°C
	LgassR	TCTTTTAAACTCTAGACATGCGTC			
L. jensenii	LjensF	AAGTCGAGCGAGCTTGCCTATAGA	15 min, 95°C	40	15 s 95°C, 55 s 60°C, 60 s 72°C
	LjensR	CTTCTTTCATGCGAAAGTAGC			
L. vaginalis	LV16s_23s_F	GCCTAACCATTTGGAGGG	15 min, 95°C	37	15 s 95°C, 30 s 56°C, 30 s 72°C
	LV16s_23s_R3	CGATGTGTAGGTTTCCG			
A. vaginae	AV-F	CCCTATCCGCTCCTGATACC	10 min, 95°C	40	15 s 95°C, 20 s 64°C, 25 s 72°C
	AV-R	CCAAATATCTGCGCATTTCA			
G. vaginalis	F-GV1	TTACTGGTGTATCACTGTAAGG	10 min, 95°C	40	45 s 95°C, 45 s 55°C, 45 s 72°C
	R-GV3	CCGTCACAGGCTGAACAGT			
M. curtisii	F	GCGATGGTTCCAGAGATGGGCCAGCCTT	2 min, 95°C	40	60 s 95°C, 60 s 65°C, 60 s 72°C
	R	CACGAGTCCCCGGCCGAA			
P. bivia	PBsulF	ACGTTTGGGCAAAGCTCCTTGTCT	1 min, 95°C	40	15 s 94°C, 40 s 58°C, 30 s 72°C
	PBsulR	GCGTGTACGCCAGTTGCAAGA			

a RT-qPCR, reverse transcription-quantitative PCR.

Absolute bacterial quantities (in cells/ml) were then calculated by comparison to a standard curve of genomic DNA from pure culture of the reference species (see “Strains, growth conditions, and prebiotics used,” above). Isolated DNA from L. iners was used as a standard for total Lactobacillus due to the common abundance of this organism in the vaginal microbiota (4). Total copies of the gene of interest in standards were calculated using total DNA content from measurements by the NanoDrop ND-1000 spectrophotometer (NanoDrop Technologies, Inc., Wilmington, DE) and known gene copies per genome were determined by searching the organism in the NCBI Genome database. Nondetects were treated as containing no DNA and averaged with concentrations of other replicates to reduce positive bias (55).

### Targeted gas chromatography-mass spectrometry.

To precipitate proteins, 100 μl of supernatant from swabs grown in prebiotics (above) was diluted with 200 μl of 1:1 methanol:H_2_O, vortexed for 10 s, and then centrifuged for 10 min at 10,000 × *g*. Half (150 μl) of the supernatant was collected into a vial, along with 2 μl of 1 mg/ml ribitol standard, where it was dried in a SpeedVac without heat. Samples were derivatized with 50 μl of 2% methoxyamine-HCl in pyridine (MOX) at 50°C for 90 min, followed by 50 μl of *N*-methyl-*N*-(trimethylsilyl)-trifluoroacetamide (MSTFA) at 50°C for 30 min. To prepare for injection, samples were transferred into microinserts.

One μl of sample was injected into an Agilent (Santa Clara, CA) 7890A gas chromatograph (GC)/5975c inert mass selective detector (MSD) with a triple axis detector. Samples were injected using pulsed splitless mode using a 30-m DB5-MS column with 10-m DuraGuard, diameter 0.35 mm, thickness 0.25 μm (J&W Scientific, Folsom, CA). Helium was used as the carrier gas at a constant flow rate of 1 ml/min. Oven temperature was held at 70°C for 5 min, and then increased at a rate of 5°C/min to 300°C and held for 10 min. Solvent delay was set to 7 min, and total run time was 61 min. Masses between 25 *m/z* and 600 *m/z* were selected by the detector. Samples were run in random order, and one specific sample was included in every batch as a quality control for machine consistency.

Chromatogram files were deconvoluted and converted to .elu format using AMDIS mass spectrometry software (56) with the sensitivity set to low, resolution to medium, and support threshold to high. Chromatograms were aligned using SpectConnect (spectconnect.mit.edu) (57) with the support threshold set to low. The integrated signal (IS) matrix output was used for all further analysis. Zeros were replaced with two-thirds the minimum detected value on a per-metabolite basis followed by a log base 2 transformation. All further analyses were performed using these log-transformed values. Identities of known metabolites of interest were confirmed by comparison to standards.

### Targeted liquid chromatography-mass spectrometry.

Due to poor separation of lactate peaks using GC-MS, liquid chromatography was used to quantify lactate. Samples were vortexed for 15 s, then transferred to microinserts and directly injected into an Agilent 1290 Infinity high-pressure liquid chromatograph (HPLC) coupled to a Q-Exactive Orbitrap mass spectrometer (Thermo-Fisher) with a heated electrospray ionization (HESI) source. For HPLC analysis, 2 μl of each sample was injected into a Zorbax Eclipse Plus C18 2.1 × 50 mm × 1.6 μm column. Mobile phase A consisted of 0.1% formic acid in water and mobile phase B consisted of 0.1% formic acid in acetonitrile. The initial composition of 0% (B) was held constant for 30 s and increased to 100% over 3.0 min. Mobile phase B was then held at 100% for 1.5 min and returned to 0% over 30 s for a total runtime of 6 min.

Full MS scanning between the ranges of *m/z* 50 and 750 was performed on all samples in negative mode at 140,000 resolution. The HESI source was operated under the following conditions: nitrogen flow of 25 and 15 arbitrary units for the sheath and auxiliary gas, respectively; probe temperature and capillary temperature of 425°C and 260°C, respectively; and spray voltage of 3.9 kV. The automatic gain control (AGC) target and maximum injection time were 3 × 10^6^ and 500 ms, respectively. For molecular characterization, every tenth sample was also analyzed with a data-dependent MS/MS method where a 35,000-resolution full MS scan identified the top 12 signals above a threshold of 8.3 × 10^4^, which were subsequently selected at a 1.2 *m/z* isolation window for MS/MS. Normalized collision energy for MS/MS was 28, resolution 17,500, AGC target 10^5^. and maximum injection time was 60 ms. Blanks of pure methanol and water were run between every sample to limit carryover, and a single sample was run multiple times with every batch to account for sample variation. After data acquisition, Thermo RAW files were converted to mzML format and centroided using ProteoWizard (58). Files were then imported into R using the XCMS package (59) for chromatogram alignment and deconvolution. Features were detected with the xcmsSet function, using the centWave method and a 1 ppm tolerance. Prefilter was set to 3 to 5,000, noise to 10^3^, and signal-to-noise threshold to 5 in negative mode. Retention time correction was conducted using the obiwarp method, grouping included features present in at least 25% of samples, allowable retention time deviation was 5 s, and *m/z* width was set to 0.015. Areas of features below the signal-to-noise threshold in the data were integrated using the fillPeaks function with default settings. Any remaining zeros in the data were then replaced with two-thirds the minimum value on a per-mass basis before log base 2 transformation. The log-transformed mass list was then exported as a single txt file and used for all further analyses. For principal-component analysis, data were Pareto scaled using the MetabolAnalyze package in R. Principal components were then plotted using the pca function from the FactoMineR package in R. Identities of metabolites were determined by authentic standards based on accurate mass, retention time, and MS/MS spectra.

### Statistical analyses.

Growth curves and pH bar plots were graphed and statistically analyzed using GraphPad Prism 6 (La Jolla, CA). Significantly enhanced growth compared to the control media without prebiotic was identified using 2-way repeated measures analysis of variance (ANOVA) with Dunnett's multiple comparison test. The Kruskal-Wallis test with Dunn's multiple comparison test was used to identify differences in pH compared to the control media without prebiotic. Figure 3 was prepared using R.

### Ethics approval and consent to participate.

Ethics approval for collection of human samples was obtained from Western University's Health Science Research Ethics Board (HSREB). Written informed consent was obtained from each participant.

## Supplementary Material

Supplemental material
