# Supplementary material for: Promising Prebiotic Candidate Established by Evaluation of Lactitol, Lactulose, Raffinose, and Oligofructose for Maintenance of a Lactobacillus-Dominated Vaginal Microbiota
Source: Appl Environ Microbiol. 2018 Feb 14;84(5):e02200-17. doi: 10.1128/AEM.02200-17 (PMC5812932; doi:10.1128/AEM.02200-17)
Supplement: Supplemental material [file supp_84_5_e02200-17__index.html]

Supplemental material 

# Promising Prebiotic Candidate Established by Evaluation of Lactitol, Lactulose, Raffinose, and Oligofructose for Maintenance of a Lactobacillus-Dominated Vaginal Microbiota

## Supplemental material

- Supplemental file 1 -

  Lactulose ubiquitously utilized by vaginal *L. crispatus* clinical isolates (Fig. S1); abundance of various BV organisms from a vaginal swab consortium grown in prebiotics (Fig. S2); abundance of lactitol, lactulose, and raffinose following vaginal swab consortium growth (Fig. S3).

  PDF, 2.1M
