# Supplementary material for: Promising Prebiotic Candidate Established by Evaluation of Lactitol, Lactulose, Raffinose, and Oligofructose for Maintenance of a Lactobacillus-Dominated Vaginal Microbiota
Source: Appl Environ Microbiol. 2018 Feb 14;84(5):e02200-17. doi: 10.1128/AEM.02200-17 (PMC5812932; doi:10.1128/AEM.02200-17)
Supplement: Supplemental material [file AEM.02200-17_zam005188347s1.pdf]

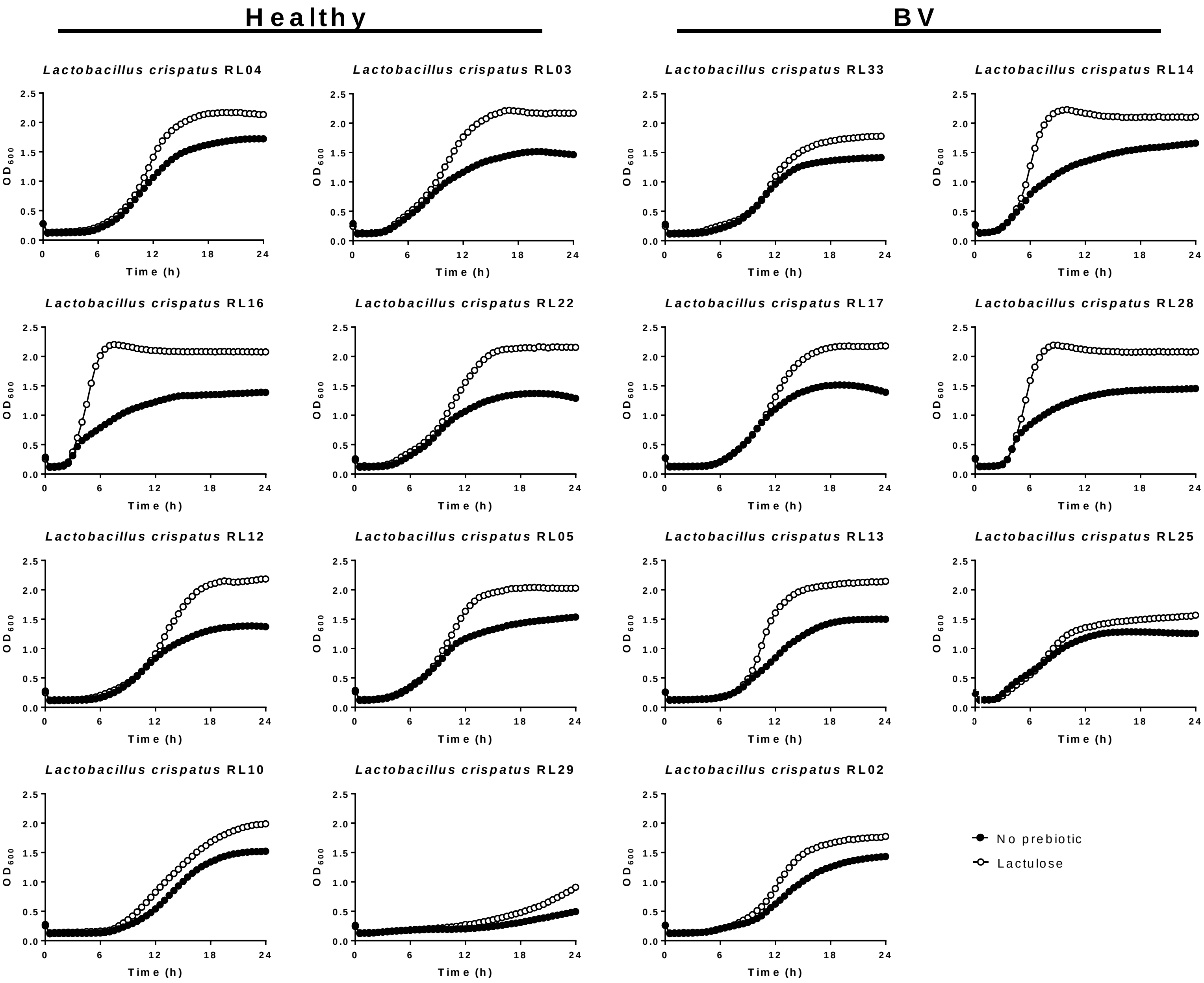

Figure S1. Lactulose is ubiquitously utilized by vaginal *L. crispatus* clinical isolates. Individual cultures of vaginal *L. crispatus* isolates obtained from women with or without BV were inoculated in dextrose-free MRS supplemented with 0.5% (w/v) lactulose. Points represent the mean $OD_{600} \pm$ SD.

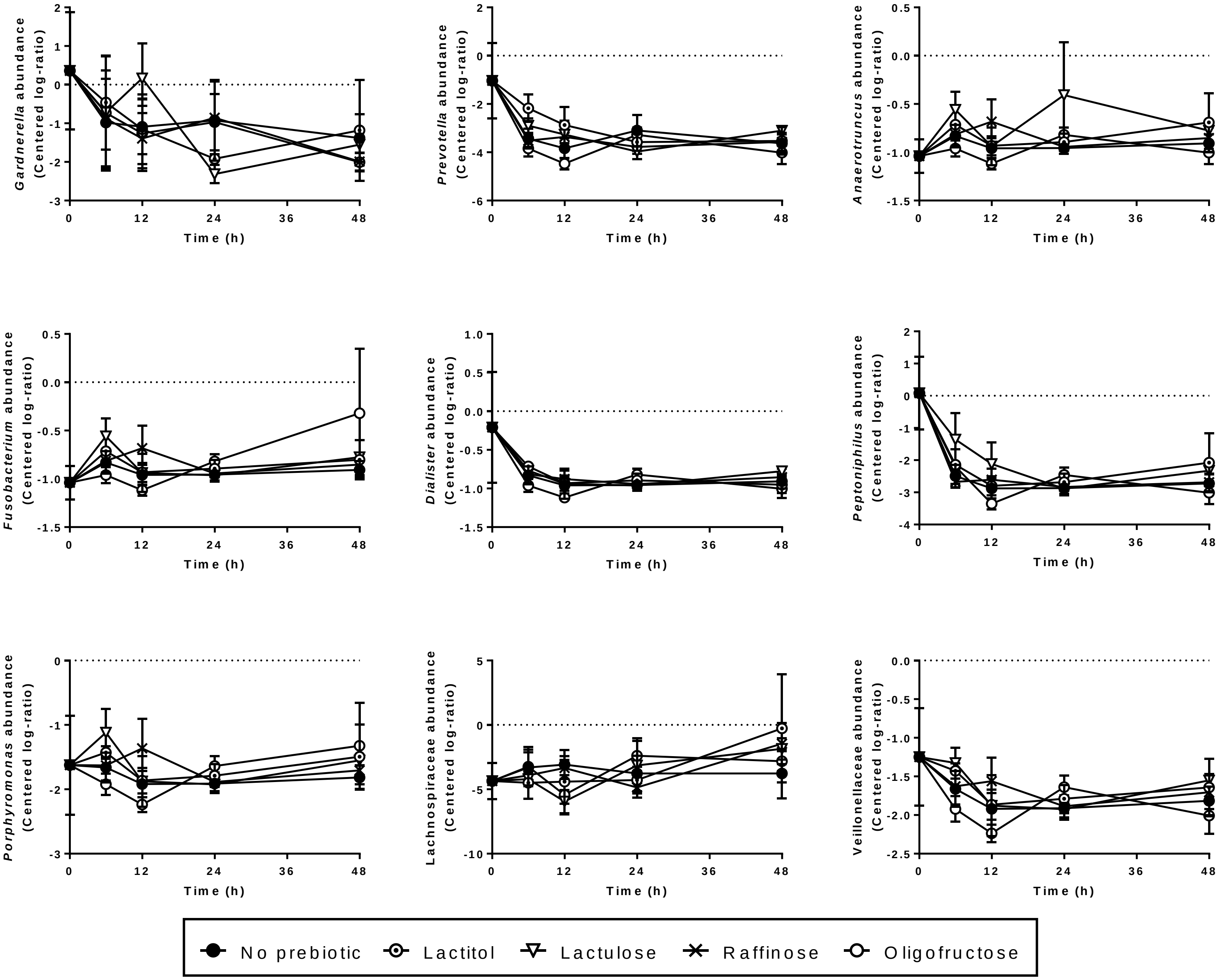

Figure S2. Abundance of various BV organisms from a vaginal swab consortium grown in prebiotics. Centered log-ratios of BV-associated microbes from 16S rRNA sequencing following growth in 0.5% prebiotics or no prebiotic control. There were no significant differences ($p>0.05$), according to two-way ANOVA with Dunnett's multiple comparisons test.

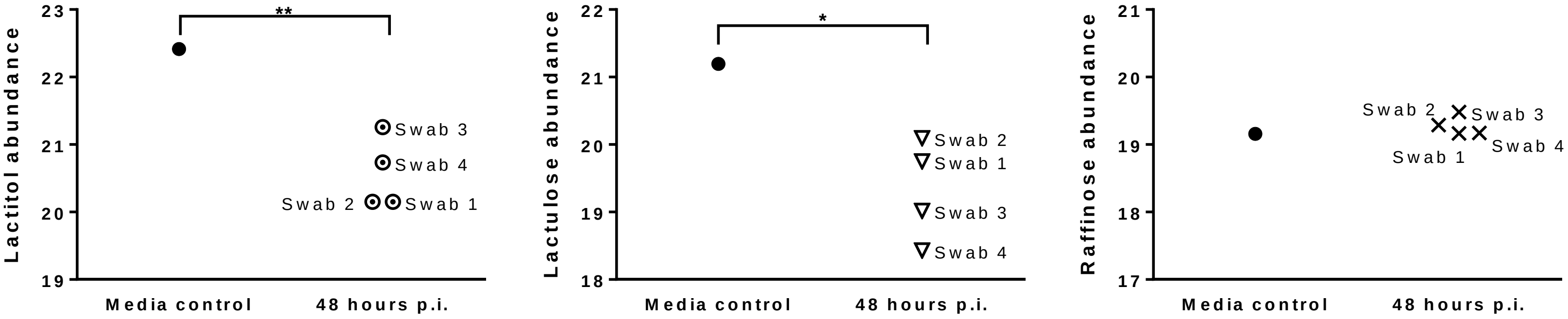

Figure S3. Abundance of lactitol, lactulose, and raffinose following vaginal swab consortium growth. Peak area of GC-MS-detected prebiotics, log2 transformed, in swab bacterial supernatant before addition of swab bacteria (media control) and after 48 hours of growth. Mean quantities are significantly different from baseline, according to the one-sample t-test (* $p<0.05$, ** $p<0.01$).
